# Genomic analysis of carbapenem-resistant non-*baumannii Acinetobacter* harboring chromosomal *bla*_OXA-23_ or plasmid-mediated *bla*_NDM-1_, *bla*_IMP-14_, and *bla*_OXA-58_

**DOI:** 10.3389/fmicb.2026.1863996

**Published:** 2026-07-27

**Authors:** Jun Sung Hong, Dokyun Kim, Seok Hoon Jeong

**Affiliations:** 1 Department of Companion Animal Health and Science, Silla University, Busan, Republic of Korea; 2 Department of Laboratory Medicine and Research Institute of Bacterial Resistance, Yonsei University College of Medicine, Seoul, Republic of Korea

**Keywords:** *Acinetobacter*, carbapenemase, community-acquired, IMP-14, OXA-23, VIM-2, NDM-1, OXA-58

## Abstract

**Introduction:**

Carbapenem-resistant *Acinetobacter* species are among the top priority pathogens, posing a threat to public health in both hospital- and community-acquired settings. Notably, the prevalence of carbapenemase-producing *non-baumanniiAcinetobacter* (CP-NBA) has recently increased worldwide.

**Methods:**

This study aimed to describe the molecular characteristics of *non-baumannii Acinetobacter* isolates with two or more carbapenemases in South Korea. Among 590 nonduplicated carbapenem-resistant *Acinetobacter* species collected from community hospitals in South Korea between 2022 and 2023, a total of 13 CP-NBA isolates were used in this study. Whole-genome sequencing and *in silico* molecular analysis were performed to characterize their genomic features.

**Results:**

Of the thirteen CP-NBA isolates, nine (four *A. nosocomialis*, two *A. pittii*, two *A. seifertii*, and one *A. bereziniae*) carried chromosomal *bla*_OXA-23_ integrated within Tn2006 in AbaRs. In addition, two *A. bereziniae* isolates with Rep3-T25 plasmids carrying *bla*_VIM-2_ and *bla*_OXA-58_ and one *A. bereziniae* isolate with the Rep3-T25 plasmid carrying *bla*_NDM-1_ and *bla*_OXA-58_ were identified. Finally, one A. *pittii* isolate with a putative 289 kb plasmid carrying three carbapenemase genes, *bla*_NDM-1_, *bla*_OXA-58_, and *bla*_IMP-14_, lacking a replication origin gene was detected.

**Conclusion:**

In conclusion, our study described the genomic structure of carbapenemase genes and related mobile genetic elements in 13 CP-NBA isolates. Although the carbapenemase genotypes are diverse, CP-NBA strains are still relatively rare in local clinics in South Korea. In particular, the *bla*_IMP-14_ gene was first identified in South Korea. These results suggest that the dissemination of CP-NBA isolates should be handled carefully in terms of transmission between bacterial strains in the clinical setting.

## 1 Introduction

The *Acinetobacter* genus contains a large number of species, including more than 80 species with validly published names^1^ and over 140 taxa with unnamed genomic species (Parte et al., 2020; Qin et al., 2021). Among these, *Acinetobacter baumannii*, *Acinetobacter nosocomialis*, and *Acinetobacter pittii* are the most prevalent, while other species such as *Acinetobacter bereziniae* and *Acinetobacter seifertii* have also been recently reported in clinical settings (Oke et al., 2024; Migliaccio et al., 2023).

*Acinetobacter* species are primarily responsible for nosocomial infections, including pneumonia and bacteremia (Wong et al., 2017). Carbapenems have been widely used to treat *Acinetobacter* infections due to their broad-spectrum antimicrobial activity (Boutzoukas and Doi, 2025). However, the acquisition of carbapenemases renders these antimicrobials ineffective, making infections caused by carbapenem-resistant isolates difficult to treat (Lee et al., 2012; Chatterjee et al., 2016). Therefore, carbapenem-resistant *Acinetobacter* species, including *A. baumannii* strains, are designated as one of the top-priority pathogens by the WHO due to limited treatment options available (Tacconelli et al., 2018).

Carbapenem-resistant non-*baumannii Acinetobacter* usually harbor metallo-β-lactamases (MBLs) located on their plasmids such as IMP, VIM, or NDM (Na et al., 2021; Jiang et al., 2019; Lee et al., 2010; Figueiredo et al., 2008; Chu et al., 2001), whereas carbapenem resistance in *A. baumannii* strains has been mainly associated with chromosomal or plasmid-mediated *bla*_OXA-23_ (Yoon et al., 2017). Recently, *Acinetobacter* species harboring chromosomal or plasmid-mediated Ambler class D *β-lactamases* (OXA-type *β-lactamases*; Poirel and Nordmann, 2006; Yamamoto et al., 2013) or class C carbapenemases such as *Acinetobacter*-derived cephalosporinases (ADCs) have been reported (Stewart et al., 2025). Although our earlier studies suggested the presence of carbapenemase-producing non-*baumannii Acinetobacter* (CP-NBA) in South Korea, a detailed molecular and genetic characterization was not performed. This study aims to investigate the molecular characteristics of resistance determinants and related mobile genetic elements in CP-NBA isolates collected from community hospitals using whole-genome sequencing.

## 2 Materials and methods

### 2.1 Bacterial strains

This study included 13 CP-NBA isolates, which were previously identified from a collection of 590 non-duplicate carbapenem-resistant *Acinetobacter* isolates obtained from patients at a long-term care facility and a local general hospital between 2022 and 2023 (Bae and Hong, 2024).

The 14 frozen CP-NBA samples were thawed and subcultured on MacConkey agar plates. Sequence analyses of the *rpoB* gene were performed to confirm precise bacterial identification. The minimum inhibitory concentrations (MICs) of carbapenems were determined by the broth microdilution method according to CLSI guidelines. In this method, each test isolate was exposed to serial 2-fold dilutions of antibiotics. Additionally, carbapenemase production was tested using modified carbapenemase inactivation methods (mCIMs), where a carbapenem disk is incubated with the test isolate and subsequently placed on *Escherichia coli* ATCC25922 lawn (Tsai et al., 2020). In addition, conventional PCR for carbapenemase genes, including *bla*_KPC_, *bla*_VIM_, *bla*_IMP_, *bla*_NDM_, and *bla*_OXA-23-like_, was performed to confirm their carbapenemase production. The primers used in this study are summarized in Supplementary Table S1.

### 2.2 Whole-genome sequencing and *in silico* molecular analysis

Genomic DNA was extracted from the 14 isolates using a GenElute Bacterial Genomic DNA Kit (Sigma-Aldrich, St. Louis, MO, USA). The DNA was sheared with a g-TUBE apparatus (Covaris, Inc., Woburn, MA), and the fragments were purified by using 0.45x volume of Agencourt AMPure XP magnetic beads (Beckman Coulter, Inc., Brea, CA) as previously described (Hong et al., 2016). Whole-genome sequencing (WGS) was performed by single-molecule real-time (SMRT) sequencing with a 1 M SMRT cell on a PacBio Sequel II instrument (PacBio, Menlo Park, CA). Microbial assembly of the raw sequence reads was performed using PBMM2 (https://github.com/PacificBiosciences/pbmm2; last updated in December 2025, accessed on 16 January 2026). The coding, tRNA, and rRNA sequences were annotated using the NCBI Prokaryotic Genome Annotation Pipeline (Seemann, 2014).

Plasmids were typed using AcinetobacterPlasmidTyping V3.0 (https://github.com/MehradHamidian/AcinetobacterPlasmidTyping; accessed on 24 May 2026). Resistance genes were analyzed using ResFinder with thresholds of 90% nucleotide identity (https://genepi.food.dtu.dk/resfinder/; accessed on 16 January 2026) from the Center for Genomic Epidemiology server. The circular plasmid maps were generated using the Proksee online tool for visualization purposes only, with default parameters.^2^ The genetic context of *bla*_OXA-23_, including the presence of upstream IS*Aba1* and its association with the Tn*2006* and Tn*2009* composite transposons, was analyzed using the ISfinder database with default BLAST parameters.^3^

### 2.3 Accession numbers

The whole-genome project of 13 CP-NBA strains has been deposited at GenBank. JBTLOO000000000 for EO2022_ACS-73 under the BioProject PRJNA1398012, JBTLOP000000000 for EO2022_ACS-203 under the BioProject PRJNA1398013, JBTNLF000000000 for SC2023_ACS-205 under the BioProject PRJNA1398435, JBTNLC000000000 for SC2023_ACS-220 under the BioProject PRJNA1398437, JBTNLG000000000 for SC2023_ACS-226 under the BioProject PRJNA1398438, JBTNLD000000000 for SC2023_ACS-228 under the BioProject PRJNA1398439, JBTNLE000000000 for SC2023_ACS-251 under the BioProject PRJNA1398458, JBTNLI000000000 for SC2023_ACS-366 under the BioProject PRJNA1398497, JBTNLJ000000000 for SC2023_ACS-392 under the BioProject PRJNA1398501, JBTNLK000000000 for SC2023_ACS-419 under the BioProject PRJNA1398505, JBTNLL000000000 for SC2023_ACS-444 under the BioProject PRJNA1398506, JBTNLM000000000 for SC2023_ACS-445 under the BioProject PRJNA1398508, JBTNLN000000000 for SC2023_ACS-472 under the BioProject PRJNA1398515, and JBZDOF000000000 for SC2023_ACS-253 under the BioProject PRJNA1475936.

## 3 Results

### 3.1 Characteristics of CP-NBA

WGS was performed on 14 CP-NBA isolates identified as carbapenemase producers by mCIM and conventional PCR. However, *bla*_NDM-1_ was not detected in the genome of one *A. bereziniae* isolate (SC2023_ACS-253) despite its initial positivity both by mCIM and *bla*_NDM-1_ PCR, with imipenem and meropenem MICs of 8 μg/mL. On repeat testing, this isolate showed imipenem and meropenem MICs of 4 μg/mL and was negative by both mCIM and *bla*_NDM-1_ PCR, indicating that the initial carbapenemase-positive results were not reproducible, suggesting the initial error or plasmid loss. Therefore, this isolate was excluded, and the remaining 13 CP-NBA isolates were finally included in this study. Genomic analysis of SC2023_ACS-253 revealed *bla*_OXA-229_ on the chromosome and eight plasmids ranging from 6 to 74 kb, with a *bla*_MCA-like_ gene carried on the second-largest plasmid (34 kb). This *bla*_MCA-like_ gene showed 92.3% nucleotide identity (1,091/1,173 bp) to *bla*_MCA-1_ (LMZW01000049.1). The sequence data were deposited in NCBI GenBank under accession number PX854773.

A total of 13 CP-NBA isolates originating from urine or sputum were identified in 9 community hospitals in 7 regions of South Korea (Table 1). Nine *Acinetobacter* species harboring the *bla*_OXA-23_ gene, including four *A. nosocomialis*, two *A. pittii*, two *A. seifertii*, and one *A. bereziniae*, were identified. In the remaining four CP-NBA isolates, the coexistence of *bla*_VIM-2_ and *bla*_OXA-58_ in two *A. bereziniae* isolates (SC2023_ACS-205 and SC2023_ACS-444), that of *bla*_NDM-1_ and *bla*_OXA-58_ in one *A. bereziniae* isolate (SC2023_ACS-472), and that of *bla*_NDM-1_, *bla*_OXA-58_, and *bla*_IMP-14_ in one *A. pittii* isolate (SC2023_ACS-251) were identified.

**Table 1:** Molecular profiles of 13 carbapenemase-producing non-*Baumannii Acinetobacter* isolates.

Isolate	Strain	Hospital (region)	Sample	Carbapenemase	Location	Plasmid rep type	Coresistance gene	MIC (μg/mL)	
								IPM	MEM
EO2022_ACS-73	API	I (G)	Urine	OXA-23	Chromosome	-	*ant(2″)-Ia*, *bla*_ADC-25_, *bla*_OXA-500_	32	32
EO2023_ACS-203	API	II (D)	Sputum	OXA-23	Chromosome	-	*ant(2″)-Ia*, *bla*_ADC-25_, *bla*_OXA-500_	32	32
SC2023_ACS-205	ABZ	III (B)	Urine	VIM-2 + OXA58	Plasmid	Rep3-T25	*aph(3′)-*Via, *aac(6′)-II*, *aph(6)-Id*, *aph(3″)-Ib*, *aadA1*, *sul1*, *sul2*, *msr(E)*, *mph(E)*	16	8
SC2023_ACS-220	ABZ	IV (G)	Urine	OXA-23	Chromosome	-	*aph(6)-Id*, *aph(3″)-Ib*, *bla*_OXA-257_, *sul2*, *tet(39)*	16	16
SC2023_ACS-226	ASE	V (JN)	Sputum	OXA-23	Chromosome	-	*aph(6)-Id*, *aph(3″)-Ib*, *bla*_ADC-25_, *sul2*, *msr(E)*, *mph(E)*, *tet(39)*	32	32
SC2023_ACS-228	ANS	VI (CB)	Sputum	OXA-23	Chromosome	-	*ant(2″)-Ia*, *bla*_ADC-25_	32	32
SC2023_ACS-251	API	VII (G)	Urine	NDM-1 + IMP-14 + OXA-58	Plasmid	No rep	*aph(3′)-VIa*, *ant(2″)-Ia*, *aac(6′)-Ib3*, *aph(6)-Id*, *aph(3″)-Ib*, *aadA2b*, *qnrVC6*, *bla*_ADC-25_, *sul1*, *msr(E)*, *mph(E)*, *cmlA1*, *floR*	128	64
SC2023_ACS-366	ANS	VI (CB)	Sputum	OXA-23	Chromosome	-	*ant(2″)-Ia*, *bla*_ADC-25_	32	32
SC2023_ACS-392	ANS	VI (CB)	Sputum	OXA-23	Chromosome	-	*ant(2″)-Ia*, *bla*_ADC-25_	32	32
SC2023_ACS-419	ANS	VI (CB)	Sputum	OXA-23	Chromosome	-	*ant(2″)-Ia*, *bla*_ADC-25_	32	32
SC2023_ACS-444	ABZ	VIII (JB)	Sputum	VIM-2 + OXA58	Plasmid	Rep3-T25	*aph(3′)-*Via, *aac(6′)-Il*, *aph(6)-Id*, *aph(3″)-Ib*, *aadA1*, *sul1*, *sul2*, *msr(E)*, *mph(E)*	16	8
SC2023_ACS-445	ASE	V (JN)	Sputum	OXA-23	Chromosome	**-**	*aph(6)-Id*, *aph(3″)-Ib*, *bla*_ADC-25_, *sul2*, *msr(E)*, *mph(E)*, *tet(39)*	32	32
SC2023_ACS-472	ABZ	IX (KB)	Urine	NDM-1 + OXA-58	Plasmid	Rep3-T25	*aac(3)-IId*, *aph(3′)-*Via, *aph(6)-Id*, *aph(3″)-Ib*, *bla*_OXA-257_, *sul2*, *tet(39)*	64	32

ACS, *Acinetobacter* species; API, *Acinetobacter pittii*; ABZ, *Acinetobacter bereziniae*; ASE, *Acinetobacter seifertii*; ANS, *Acinetobacter nosocomialis*; G, Gyeonggi; D, Daegu; B, Busan; JN, Jeonnam; CB, Chungbuk; JB, Jeonbuk; KB, Gyeongbuk.

The carbapenem MICs for the 13 CP-NBA isolates ranged from 16 to 128 μg/mL for imipenem and from 8 to 64 μg/mL for meropenem (Table 1), indicating resistance to carbapenems. *A. pittii* isolate (SC2023_ACS-251), which carried *bla*_NDM-1_, *bla*_IMP-14_, and *bla*_OXA-58_, had the highest MIC values (128 μg/mL and 64 μg/mL for imipenem and meropenem, respectively).

### 3.2 Genetic environments of OXA-23-producing non-*baumannii Acinetobacter*

**Figure 1:**
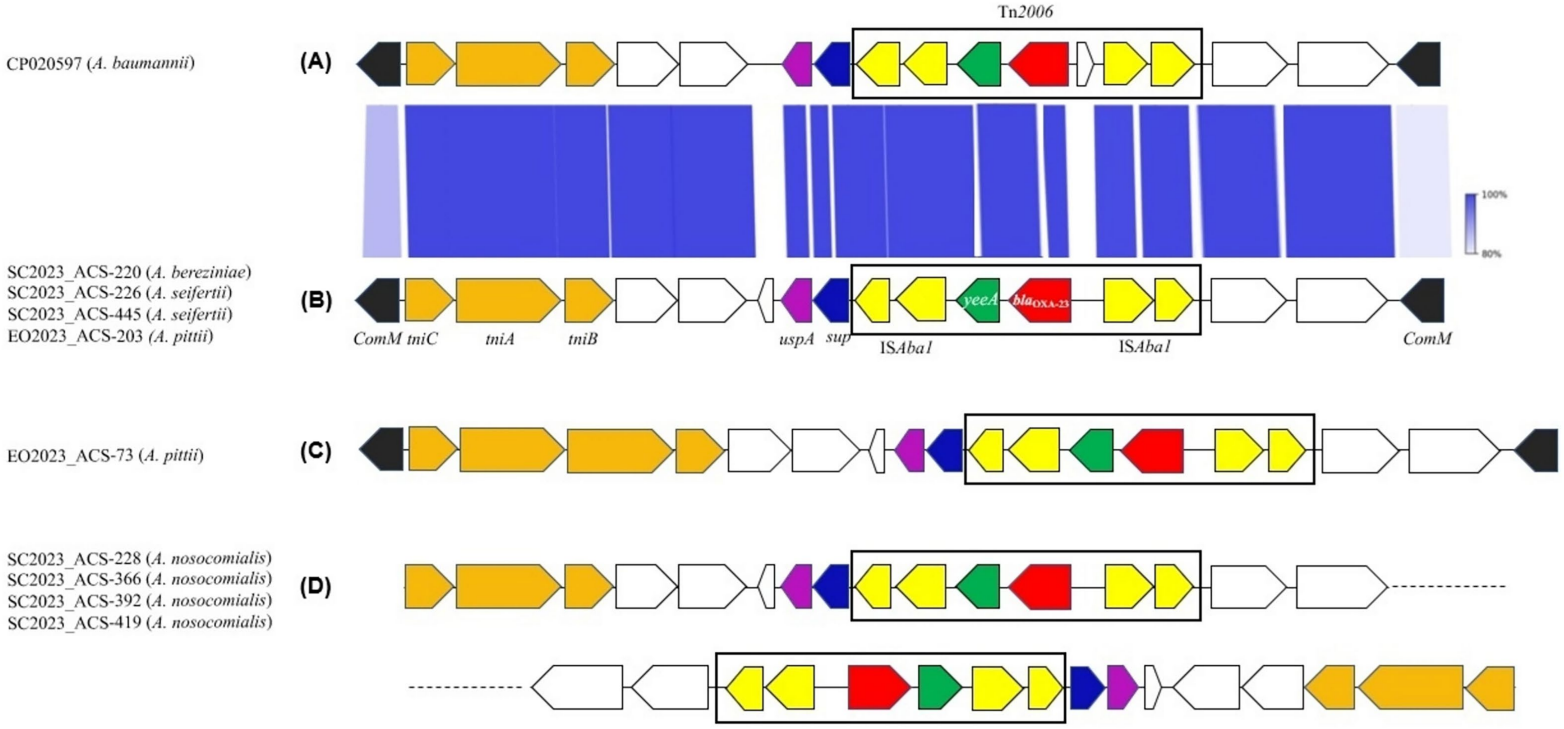
Genetic contexts of carbapenemase OXA-23 associated with AbaRs, including Tn*2006*. The strains and *Acinetobacter* species names are represented on the left. **(A)** AbaR4 in *A. bauman* nii strain HWBA8. **(B)** Four CP-NBA isolates harboring ca. 18 kb of the AbaR. **(C)** One *A. pittii* isolate harbored AbaR. **(D)** Four *A. nosocomialis* isolates harboring an extra copy of AbaR.

A total of 9 CP-NBA isolates, including four *A. nosocomialis*, two *A. pittii*, two A. *seifertii*, and one *A. bereziniae* were analyzed for further genetic environments of transposons harboring the *bla*_OXA-23_ gene on chromosomes ranging from 3.80 to 4.49 Mb (Table 1). The *bla*_OXA-23_ gene was carried by a chromosomally integrated Tn*2006*, which contained two IS*Aba1* copies and only the *yeeA* gene encoding a DNA methylase, in all nine CP-NBA isolates (Figure 1). In addition, two to seven acquired AMR genes were identified on each chromosome: *ant(2″)-Ia*, *aph(6)-Id*, *aph(3″)-Ib*, *bla*_ADC-25_, *bla*_OXA-257_, *bla*_OXA-500_, *sul2*, *msr(E)*, *mph(E)*, and/or *tet(39)* (Table 1). The integration site of Tn*2006*s, associated with *A. baumannii* resistance islands (AbaRs), was equally at the *sup* gene for sulfate permease among all nine CP-NBA isolates. In this study, two AbaR composite genes, integrated at the *comM* gene encoding ATPase, were identified in 5 CP-NBA isolates (*A. bereziniae* SC2023_ACS-220, *A. seifertii* SC2023_ACS-226, *A. seifertii* SC2023_ACS-445, *A. pittii* EO2023_ACS-73, and *A. pittii* EO2023_ACS-203; Figures 1B,C). Notably, two *bla*_OXA-23_ gene copies were detected in the remaining four CP-NBA isolates (*A. nosocomialis* SC2023_ACS-228, *A. nosocomialis* SC2023_ACS-366, *A. nosocomialis* SC2023_ACS-392, and *A. nosocomialis* SC2023_ACS-419; Figure 1D). They possessed an identical extragenetic environmental copy in opposite orientation, which is not associated with the *comM* gene.

### 3.3 Coexistence of *bla*_MBLs_ and *bla*_OXA-58_ on the plasmid

Four CP-NBA isolates (SC2023_ACS-205, SC2023_ACS-251, SC2023_ACS-444, and SC2023_ACS-472) were found to carry carbapenemase genes on plasmids via WGS. The co-existence of AMR genes on the four carbapenemase-carrying plasmids is summarized and visualized using a heatmap in Supplementary Figure S1.

**Figure 2:**
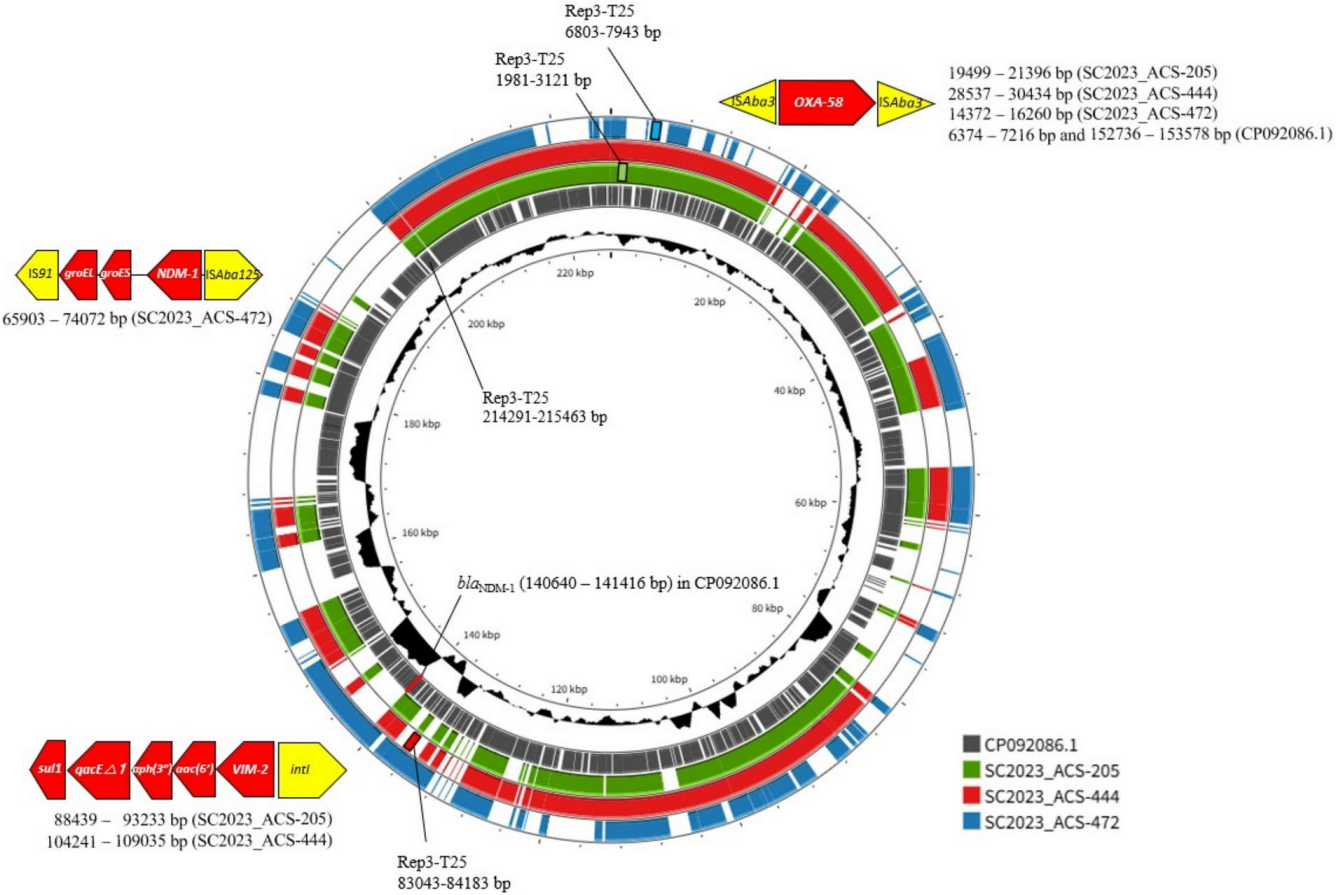
Rep3-T25 plasmid map and carbapenemase genetic environments of three CP-NBA isolates (SC2023_ACS-205, SC2023_ACS-444, and SC2023_ACS-472) compared with that of the *A. bereziniae* strain GD03393 plasmid, which was unnamed and carried NDM-1 and two copies of OXA-58 (GenBank CP092086.1) via Proksee (https://proksee.ca).

The genome of the *A. bereziniae* SC2023_ACS-205 strain comprised a 4.5-Mb circularized chromosome with three plasmids, including one mega-plasmid (p*SCAB205_VIM-2*, ca. 111 kb) carrying 123 CDSs and two small plasmids (ca. 13 kb and ca. 6 kb) lacking AMR genes. In the chromosomal contig, co-resistance genes for aminoglycosides, streptomycin, and erythromycin: *aph(6)-Id*, *aadA1*, *msr(E)*, and *mph(E)* were identified (Table 1). The *bla*_VIM-2_ gene identified in the 111 kb mega-plasmid was located on the class 1 integron as a gene cassette (InVIM-2; *intI1* – *bla*_VIM-2_ – *aac(6′)* – *aph(3″)* – *qacEΔ1* – *sul1)* within 88,439–93,233 bp (Figure 2). The *bla*_OXA-58_ gene identified in the 111 kb mega-plasmid was bracketed by IS*Aba3* (Figure 2).

**Figure 3:**
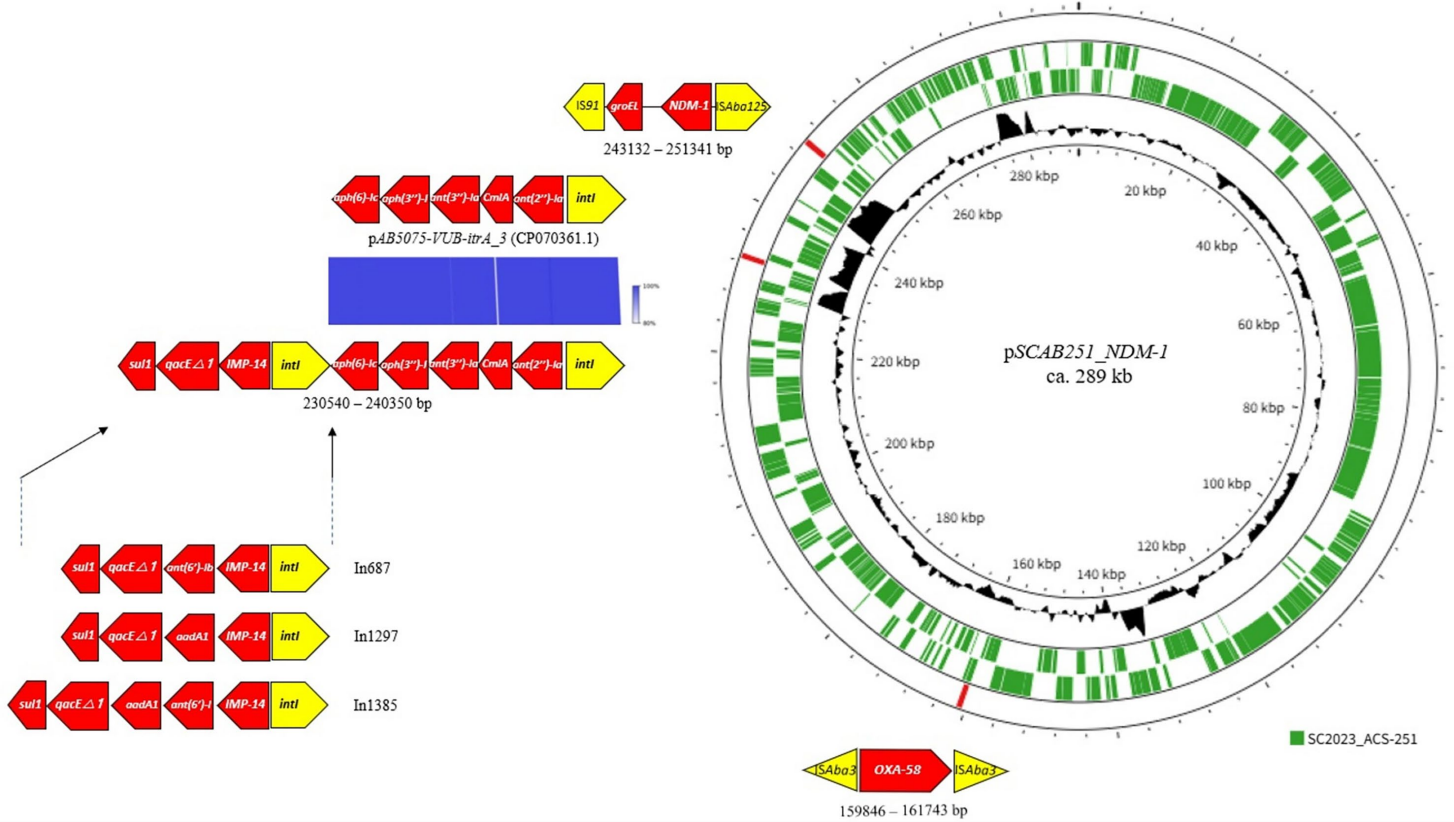
No-rep plasmid map and carbapenemase genetic environments of CP-NBA (SC2023_ACS-251) with carbapenemases NDM-1, OXA-58, and IMP-14 via Proksee (https://proksee.ca).

The genome of the *A. pittii* SC2023_ACS-251 strain comprised a 3.98-Mb circularized chromosome, one mega-plasmids (ca. 289 kb), and one small plasmid (ca. 12 kb). The presence of *qnrVC6* for ciprofloxacin resistance, *bla*_ADC-25_ for cephalosporin resistance, *msr(E)* and *mph(E*) for erythromycin resistance, and *floR* for chloramphenicol resistance was detected on the chromosome (Table 1). Three carbapenemase genes, including *bla*_NDM-1_, *bla*_IMP-14_, and *bla*_OXA-58_, which were identified in the mega-plasmid (p*SCAB251_NDM-1*), contained 297 CDSs. The *bla*_NDM-1_ gene was flanked by IS*Aba125* along with IS*91* within 243,132–251,341 bp, showing IS*Aba125* – *bla*_NDM-1_ – *groEL* – *IS91*. Notably, the SC2023_ACS-251 strain was found to carry a class 1 integron (InIMP-14; *intI1 – ant(2″)-la* – *cmlA* – *ant(3″)-Ia* – *aph(3″)-I* – *aph(6)-Ic* – *intI1 – bla_IMP-14_* – *qacEΔ1* – *sul1*) between bp 230,540 and 240,350 (Figure 3), which was not identical to any of the class I integrons in the NCBI database. The *bla*_OXA-58_ gene was bracketed by IS*Aba3* (Figure 3).

The genome of the *A. bereziniae* SC2023_ACS-444 strain has a 4.6-Mb circularized chromosome, one mega-plasmid (p*SCAB444_VIM-2*, ca. 136 kb), and a medium-sized plasmid (ca. 22 kb). p*SCAB444_VIM-2* contained 152 CDSs with *bla*_VIM-2_ and *bla*_OXA-58_. The co-resistance genes in the chromosomal contig were identical to those on the chromosome of the SC2023_ACS-205 strain (Table 1). The genetic environment of the *bla*_VIM-2_ gene within 104,241*–*109,035 bp and the *bla*_OXA-58_ gene within 28,537–30,434 bp was identical to that found *in the* SC2023_ACS-205 strain (Figure 2).

The genome of the *A. bereziniae* SC2023_ACS-472 strain consisted of nine contigs, including four contigs (total: 4.1 Mb) containing ribosomal protein genes and was considered to represent the chromosome and five plasmids. The chromosomal contigs contained *aac(3)-IId*, *aph(3′)-VIa*, *aph(6)-Id*, and *aph(3″)-Ib* for aminoglycosides; *bla*_OXA-257_ for *β*-lactam; *sul2* for sulfamethoxazole; and *tet(39)* for tetracycline (Table 1). The largest plasmid (p*SCAB472_NDM-1*, ca. 88 kb) included 98 CDSs with *bla*_NDM-1_ and *bla*_OXA-58_. The remaining four plasmids were all small plasmids (< 10 kb) without AMR genes. The *bla*_NDM-1_ gene was bracketed by IS*Aba125* along with IS*91* (IS*Aba125* – *bla*_NDM-1_ – *groES* – *groEL* – *IS91*) within 65,903–74,072 bp (Figure 2), and the *bla*_OXA-58_ gene was bracketed by IS*Aba3*, identical to that found in SC2023_ACS-205, SC2023_ACS-251, and SC2023_ACS444 (Figures 2, 3).

### 3.4 Plasmid typing for *Acinetobacter* species

Through the AcinetobacterPlasmidTyping scheme (accessed on 24 May 2026), which queries a database of *Acinetobacter* plasmid *parA*, *mob*, and *rep* sequences, three plasmids, known as Rep3-T25, were assigned to the Rep_3 subgroup (accession no. NZ_CP092086.1; Supplementary Table S2): p*SCAB205_VIM-2* (positions 1981–3,121 bp), p*SCAB444_VIM-2* (83,043–84,183 bp), and p*SCAB472_NDM-1* (6,803–7,943 bp; Figure 2). One plasmid (p*SCAB251_NDM-1*), as a no-rep group, was not assigned to an identifiable Rep protein but encoded par13-T2 and mobQ-T10. This no-rep plasmid contig (p*SCAB251_NDM-1*) was assigned as a plasmid of Proteobacteria through PlasFlow (Supplementary Table S3), and showed similarity to that of p*AHTJR1* (accession no. CP038010.1) of *Acinetobacter haemolyticus* strain TJR01 (Supplementary Table S4), containing the repB family by MOB-typer; however, a replication initiation gene of this plasmid is still lacking on the comparison map (Supplementary Figure S2).

## 4 Discussion

The AMR of *Acinetobacter* species is usually multidrug-resistant because of AMR genes on chromosomes or diverse mobile genetic elements, such as transposons, plasmids, and genomic islands (Noel et al., 2022). Particularly in carbapenem-resistant *A. baumannii*, the dominance of the carbapenemase OXA-23 has been frequently reported across continents (Boutzoukas and Doi, 2025; Yoon et al., 2017). However, the *bla*_OXA-23_ gene is rarely detected in carbapenemase-producing non-*baumannii Acinetobacter* (CP-NBA; Poirel and Nordmann, 2006; Yamamoto et al., 2013). On the other hand, the CP-NBA strain has been reported to possess diverse carbapenemases, primarily the plasmid-mediated carbapenemases *bla*_NDM-1_, *bla*_VIM-2_, and *bla*_OXA-58_, which are seldom found in carbapenem-resistant *A. baumannii* (Na et al., 2021; Jiang et al., 2019; Lee et al., 2010; Figueiredo et al., 2008; Rana et al., 2025; Yang and Rui, 2016). In this study, among the 590 carbapenem-resistant *Acinetobacter* species collected from the urine and sputum of patients in community hospitals in our previous study (Bae and Hong, 2024), nine CP-NBA isolates were identified, including four *A. nosocomialis*, two *A. pittii*, two *A. seifertii*, and one *A. bereziniae* harboring *bla*_OXA-23_ on a chromosome, were detected. Especially, plasmid-mediated *bla*_VIM-2_ and *bla*_OXA-58_ were detected in two *A. bereziniae* isolates, and plasmid-mediated *bla*_NDM-1_ and *bla*_OXA-58_ were identified in one *A. bereziniae* isolate. Finally, one *A. pittii* isolate, which contained a plasmid carrying *bla*_NDM-1_, *bla*_OXA-58_, and *bla*_IMP-14_, was identified as a novel case.

The identification of *bla*_OXA-23_ was usually less common in non-*baumannii Acinetobacter* strains than in *A. baumannii* strains. In South Korea, the *bla*_OXA-23_ gene was detected in 98% (*n* = 309) of 314 carbapenem-resistant *A. baumannii* strains in a nationwide surveillance study in 2017 (Yoon et al., 2017). Similarly, in a 2019 global survey, 81.8% (1,918 of 2,345) of *A. baumannii* genomes deposited in the NCBI GenBank database that harbored carbapenemase genes were also identified as carrying *bla*_OXA-23_ (Hamidian and Nigro, 2019). In this study, among 590 carbapenem-resistant *Acinetobacter* species, only nine CP-NBA isolates harboring the *bla*_OXA-23_ gene were identified. This gene was associated with Tn*2006*, which was integrated into the *sup* gene as described in a previous study (Yoon et al., 2017). Among them, four CP-NBA isolates harboring ca. 18 kb of the *A. baumannii* resistance island (AbaR) included Tn*2006* along with AMR genes, and these genetic contexts were similar to AbaR4 in *A. baumannii* strain HWBA8 (accession no. CP020597), which originated from South Korea in 2017, except for two hypothetical proteins (Figures 1A,B). One *A. pittii* isolate (EO2023_ACS-73) also harbored AbaR with an extra *tniA* gene (Figure 1C). In the remaining four *A. nosocomialis* isolates seen as having a clonal spread, an extra copy of AbaR was identified in the opposite direction, such as in *A. nosocomialis* strain SSA3 (accession no. CP020588; Figure 1D). The conservation of highly similar Tn*2006*-containing AbaR genetic contexts across multiple OXA-23-producing non-baumannii *Acinetobacter* species suggests horizontal dissemination of this resistance island, which may have contributed to the spread of *bla*_OXA-23_ beyond *A. baumannii*.

To the best of our knowledge, *bla*_IMP-14_ has not been directly reported from clinical isolates in South Korea. After the first submission for the *bla*_IMP-14_ gene to NCBI GenBank in 2004 and the first report of IMP-14-producing *Pseudomonas aeruginosa* in Thailand in 2011 (Piyakul et al., 2012), this AMR gene has been identified as a regional endemic and nosocomial outbreak in Thailand in *P. aeruginosa*, *A. baumannii*, and *Escherichia coli* (Piyakul et al., 2012; Kansakar et al., 2011; Stoesser et al., 2016). The *bla*_IMP-14_ gene has been reported in one of three class 1 integron arrangements: In687 (*intI1*–*bla*_IMP-14_–*aacA4*–*qacEΔ1*–*sul1*), In1297 (*intI1*–*bla*_IMP-14_–*aadA1*–*qacEΔ1*–*sul1*), and In1385 (*intI1*–*bla*_IMP-14_–*aacA7*–*aadA1*–*qacEΔ1*–*sul1*). In this study, the genetic environment of *bla*_IMP-14_ was located within two class 1 integrons arranged in tandem *(intI1–ant(2″)-Ia–cmlA–ant(3″)-Ia–aph(3″)-Ib–aph(6)-Id–intI1–bla_IMP-14_–qacEΔ1–sul1)*, which is distinct from those previously described. The first class 1 integron (*intI1*–*ant(2″)-Ia*–*cmlA*–*ant(3″)-Ia*–*aph(3″)-Ib*–*aph(6)-Id*) was identical to that carried by an *A. baumannii* plasmid (p*AB5075-VUB-itrA_3*; accession no. CP070361.1 deposited in 2021; Figure 3). This finding suggests that the tandem integron structure associated with *bla*_IMP-14_ on p*SCAB251_NDM-1* may have originated through horizontal acquisition of a previously described class 1 integron rather than arising as a novel rearrangement.

Plasmids are major mobile genetic elements in *Acinetobacter* species and are well known for disseminating AMR genes across diverse species, contributing to nosocomial spread (Noel et al., 2022). *Acinetobacter* plasmid replicons are classified on the basis of their replication initiation (Rep) proteins (Lam et al., 2023): first by family (Rep3, Rep1, or RepPriCT1) and then by type, using a 95% nucleotide identity threshold. In this study, three plasmid types, including Rep3-T25, were identified among four CP-NBA isolates. The Rep3-T25 plasmids (p*SCAB205_VIM-2* and p*SCAB444_VIM-2*) co-carrying *bla*_VIM-2_ and *bla*_OXA-58_ in two *A. bereziniae* strains, another Rep3-T25 plasmid (p*SCAB472_NDM-1*) co-carrying *bla*_NDM-1_ and *bla*_OXA-58_ in an *A. bereziniae* strain, and a Rep-unidentifiable plasmid (p*SCAB251_NDM-1*) co-carrying *bla*_NDM-1_, *bla*_OXA-58_, and *bla*_IMP-14_ in an *A. pittii* strain were identified (Table 1). The Rep3-T25 plasmid is a specific type of Rep3 family commonly identified in NDM-1-producing *Acinetobacter* species. The representation of the Rep3-T25 plasmid is the *A. bereziniae* strain GD03393 unnamed plasmid carrying *bla*_NDM-1_ and two *bla*_OXA-58_ gene copies, which originated from China in 2015 (accession no. CP092086.1). At present, the coexistence of *bla*_VIM-2_ and *bla*_OXA-58_ in *Acinetobacter* species has not yet been reported in clinical isolates from South Korea, although many studies have documented the presence of these genes separately. Interestingly, p*SCAB251_NDM-1* did not include an identifiable *rep* gene, which refers to a plasmid lacking a Rep protein or having a novel one. However, this plasmid origin was supported by the GC content (39.63%), read coverage, and presence of mobilization-related genes such as par13-T2 and mobQ-T10 (Figure 3). In addition, of the 1,972 rep genes identified in *Acinetobacter* species plasmids in the AcinetobacterPlasmidTyping database, 561 remain classified as the no-rep plasmid group (Supplementary Table S5; accessed on 24 May 2026). In this regard, our findings suggest that further investigation of the remaining plasmids lacking identifiable *rep* genes is warranted.

## 5 Conclusion

In conclusion, among the 13 CP-NBA isolates, 9 harbored chromosomal *bla*_OXA-23_, 3 carried *bla*_VIM-2_ or *bla*_NDM-1_ together with *bla*_OXA-58_, and one carried *bla*_NDM-1_, *bla*_OXA-58_, and *bla*_IMP-14_. With the exception of chromosomally located *bla*_OXA-23_, all carbapenemase genes *bla*_VIM-2_, *bla*_NDM-1_, and *bla*_IMP-14_, and *bla*_OXA-58_ were plasmid-borne. To our knowledge, this is the first report of plasmid-mediated *bla*_IMP-14_ in *Acinetobacter* in South Korea, highlighting the potential for further plasmid-driven dissemination of this carbapenemase.

## Data Availability

The datasets presented in this study can be found in online repositories. The names of the repository/repositories and accession number(s) can be found at: https://www.ncbi.nlm.nih.gov/genbank/, JBTLOO000000000, JBTLOP000000000, JBTNLF000000000, JBTNLC000000000, JBTNLG000000000, JBTNLD000000000, JBTNLE000000000, JBTNLI000000000, JBTNLJ000000000, JBTNLK000000000, JBTNLL000000000, JBTNLM000000000, and JBTNLN000000000.

## Footnotes

1
https://lpsn.dsmz.de/genus/acinetobacter

2
https://proksee.ca/

3
www-is.biotoul.fr
