# Supplementary material for: Genomic analysis of carbapenem-resistant non-baumannii Acinetobacter harboring chromosomal blaOXA-23 or plasmid-mediated blaNDM-1, blaIMP-14, and blaOXA-58
Source: Front Microbiol. 2026 Jul 27;17:1863996. doi: 10.3389/fmicb.2026.1863996 (PMC13454041; doi:10.3389/fmicb.2026.1863996)
Supplement: Supplementary file 1 [file Data_sheet_1.docx]

| **Table S1.** Primers used in this study | | |  |  |
| --- | --- | --- | --- | --- |
| Target | Primer | Nucleotide Sequence | Amplicon size (bp) | |
| *bla*_IMP-type_ | IMP-F | TGAGCAATGTATCTGTATTC | 740 | |
|  | IMP-R | TTAGTTGCTTGGTTTTGATG |  |  |
| *bla*_VIM-type_ | VIM-2F | TGGTCTACATGACCGCGTCT | 766 | |
|  | VIM-2R | CGACTGAGCGATTTGTGTG |  |  |
| *bla*_NDM-type_ | NDM-F | CAATATTATGCACCCGGTCG | 726 | |
|  | NDM-R | ATCATGCTGGCCTTGGGGAA |  |  |
| *bla*_KPC-type_ | KPC-F | ATGTCACTGTATCGCCGTCT | 893 | |
|  | KPC-R | TTTTCAGAGCCTTACTGCCC |  |  |
| *bla*_OXA-23-like_ | OXA-23F | CTCTTTTTCTTTCTGGTTGTAC | 776 | |
|  | OXA-23R | CAGCTGTTTTAATGATTTCATC |  |  |
| *rpoB* | rpoB-F | GTGATAARATGGCBGGTCGT | 511 | |
|  | rpoB-R | CGBGCRTGCATYTTGTCRT |  |  |

**Figure S1.** Co-resistance AMR genes in four carbapenemase-carrying plasmids

**Figure S2.** Plasmid map, p*AHTJR1*, of *A. haemolyticus* strain TJR01 compared with that of *A. pittii* strain SC2023_ACS251 via Proksee ([https://proksee.ca](https://proksee.ca/)).
